# Portable All-in-One Electrochemical Actuator-Sensor System for the Detection of Dissolved Inorganic Phosphorus in Seawater

**DOI:** 10.1021/acs.analchem.2c05307

**Published:** 2023-02-01

**Authors:** Chen Chen, Alexander Wiorek, Alicia Gomis-Berenguer, Gaston A. Crespo, Maria Cuartero

**Affiliations:** † Department of Chemistry, School of Engineering Science in Chemistry, Biochemistry and Health, KTH Royal Institute of Technology, SE-100 44Stockholm, Sweden; ‡ UCAM-SENS, Universidad Católica San Antonio de Murcia, UCAM HiTech, Avda. Andres Hernandez Ros 1, 30107Murcia, Spain

## Abstract

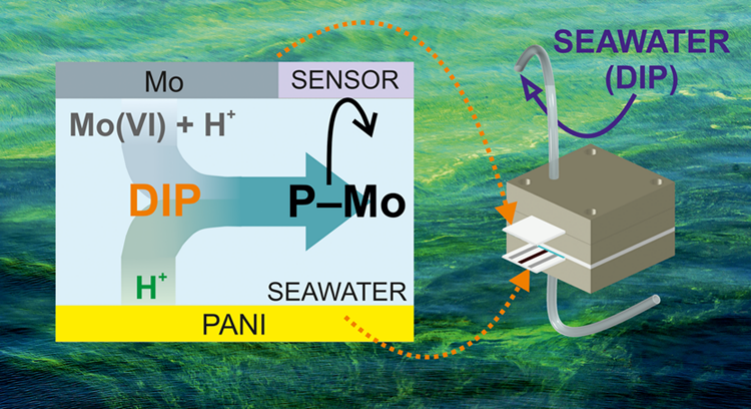

We present a methodology for the detection of dissolved inorganic phosphorous (DIP) in seawater using an electrochemically driven actuator-sensor system. The motivation for this work stems from the lack of tangible solutions for the in situ monitoring of nutrients in water systems. It does not require the addition of any reagents to the sample and works under mild polarization conditions, with the sample confined to a thin-layer compartment. Subsequent steps include the oxidation of polyaniline to lower the pH, the delivery of molybdate via a molybdenum electrode, and the formation of an electroactive phosphomolybdate complex from DIP species. The phosphomolybdate complex is ultimately detected by either cyclic voltammetry (CV) or square wave voltammetry (SWV). The combined release of protons and molybdate consistently results in a sample pH < 2 as well as a sufficient excess of molybdate, fulfilling the conditions required for the stoichiometric detection of DIP. The current of the voltammetric peak was found to be linearly related to DIP concentrations between 1 and 20 μM for CV and 0.1 and 20 μM for SWV, while also being selective against common silicate interference. The analytical application of the system was demonstrated by the validated characterization of five seawater samples, revealing an acceptable degree of difference compared to chromatography measurements. This work paves the way for the future DIP digitalization in environmental waters by in situ electrochemical probes with unprecedented spatial and temporal resolution. It is expected to provide real-time data on anthropogenic nutrient discharges as well as the improved monitoring of seawater restoration actions.

## Introduction

Phosphorous (P) is the 11^th^ most common element on earth, being essential for all living beings. Importantly, P is involved in the biogeochemical balance of the global aquatic environment, and its dissolved inorganic fraction—commonly known as DIP, primarily composed of orthophosphate—is associated with both water quality and the (un)controllable growth of algae and plankton. The massive anthropogenic discharge of P-based substances, such as fertilizers, has resulted in the undesired eutrophication of many ecosystems; this refers to the process by which an excess of nutrients results in the rapid proliferation of plant life.^1^ Although regenerative measures have been adopted and critical P limits have been set for surface waters (∼0.1 mg/L according to several environmental agencies), there are still large problems that must be overcome in certain areas, such as the Baltic Sea in the north of Europe and Mar Menor on the Spanish coast.^2,3^

The standardized method of DIP quantification in water consists of spectrophotometrically tracking the product of the reaction between orthophosphate and Mo(VI)—in the molybdate anion—in acidic and reductive media, as illustrated by eq 1.^4^
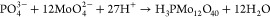
1

Because this reaction indeed occurs at a very low pH, PO_4_^3–^ and MoO_4_^2–^ species are present in their protonated forms, with H_3_PO_4_ accounting for the total DIP of the solution. Over the years, distinct readouts such as chemiluminescence, fluorescence, and atomic absorption spectrometry have been used to strengthen the analytical performance of the phosphomolybdate product chemistry.^5−7^ Together with classical chromatography strategies, these methods have been successfully utilized for the indirect detection of DIP. However, due to the inconvenience and cumbersome nature of the instruments and the need to add reagents to ensure the detectability of the analyte, these methods must be conducted in the laboratory.^8^ Alternative approaches with certain potential for decentralized analysis have been also suggested, which is especially relevant for in situ environmental sensing.^9^

It is possible to analyze P speciation (HPO_4_^2–^/H_2_PO_4_^–^) by potentiometric phosphate-selective electrodes.^10^ However, cross-selectivity and the pH dependence of the response often impede the detection of all P species. In addition, the limit of detection (LOD) is higher than the minimum requirements for unpolluted waters (traditional LODs ≥ 10 μM).^10−12^ On the other hand, once formed via the molybdate reaction described in eq 1, the phosphomolybdate complex can be reduced via electrochemistry without the need for a reducing agent in the solution.^4^ Moreover, Fogg et al. proposed the use of differential pulse voltammetry (DPV) in mixed water/organic solvents (e.g., water/acetone). Harden et al. subsequently developed an amperometry flow injection system for methanol/water solutions.^13,14^

Of the strategies that have been conducted in subsequent years, it is important to discuss the contributions of the Garçon^15−17^ and Compton groups,^11^ which represent remarkable progress regarding the electrochemical determination of DIP at the sub-micromolar level and/or to the potential development of in situ water monitoring. In a first approach, Garçon and co-workers demonstrated the formation of molybdate by sample acidification (pH ∼ 1.5) in a two-compartment electrochemical cell (Nafion-based separation principle) followed by the application of 0.05 A for 500 s, resulting in eq 2.^15^
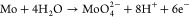
2

The phosphomolybdate complex (H_3_PMo_12_O_40_) could then be determined via amperometry or DPV, preferably through the use of a rotating gold-disk electrode.^16^ To avoid the need for convection while enhancing sensitivity as well as considering the demands of an in situ system, the concept further evolved to MoO_4_^2–^ and H^+^ delivery from the oxidation of molybdenum electrodes at 2 V, and the subsequent detection via square-wave voltammetry (SWV).^17^ Despite attaining sub-micromolar LODs, this method required long reaction times because of diffusion-dependent factors. Furthermore, different linear ranges of responses (LRRs) were obtained depending on the applied frequency in the SWV step, while side reactions could occur at the required overpotentials. These issues prevented the implementation of the concept for practical, in situ water analysis.

The key reagents required to form the phosphomolybdate complex may be delivered using ion-exchange membranes (i.e., the passive countertransport of MoO_4_^2–^ and H^+^ from an appropriate solution to the sample across membranes).^18^ An in-line flow system containing two membrane-based modules was proposed as a pretreatment for the spectrophotometric detection of DIP, resulting in a performance that was similar to a classical molybdate assay, but prospecting easier automation. In addition, paper-based sensors in which MoO_4_^2–^ and H^+^ were immobilized were developed, capable of detecting phosphates in a concentration range between 4 and 300 μM.^19^ Unfortunately, the platform was only applied to a spiked river sample.

More recently, the Compton group provided detailed evidence of the redox mechanism of the phosphomolybdate complex and further clarified the electrochemical behavior of each redox intermediate, which was essential to understand the potential avenues for reliable DIP electroanalysis.^11^ In this case, a sensor with a Mo(VI) salt immobilized in a chitosan matrix was used to generate the appropriate chemical conditions for DIP analysis, with a LOD of 0.15 μM. The analytical applicability of this concept was demonstrated for two water samples (from the tap and a pond) after the original pH of the samples was modified to 2.0. The authors also investigated the possibility of performing measurements at natural pHs to further explore the applicability of the sensor to environmental monitoring.

**Figure 1:**
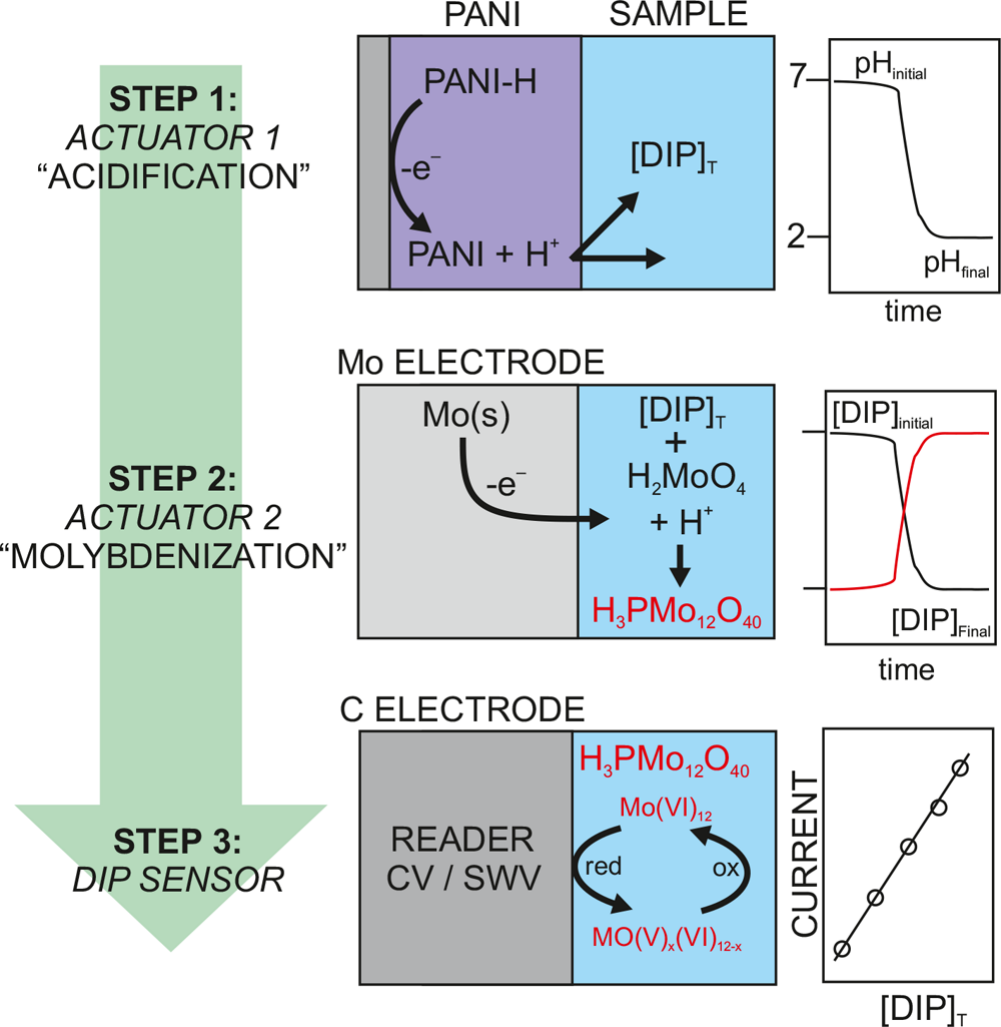
Steps involved in the detection of DIP. Step 1 acidification based on PANI oxidation via actuator 1; Step 2 the release of soluble molybdate species and protons from the molybdenum electrode via actuator 2; Step 3 the electroanalytical measurement of the phosphomolybdate complex on a screen-printed carbon electrode, [DIP]_T_ = total dissolved inorganic phosphorous; PANI = polyaniline; s = solid; CV = cyclic voltammetry; SWV = square-wave voltammetry; red = reduction; ox = oxidation; x = number of Mo(VI) centers that are reduced to Mo(V) in the detection principle.

**Figure 2:**
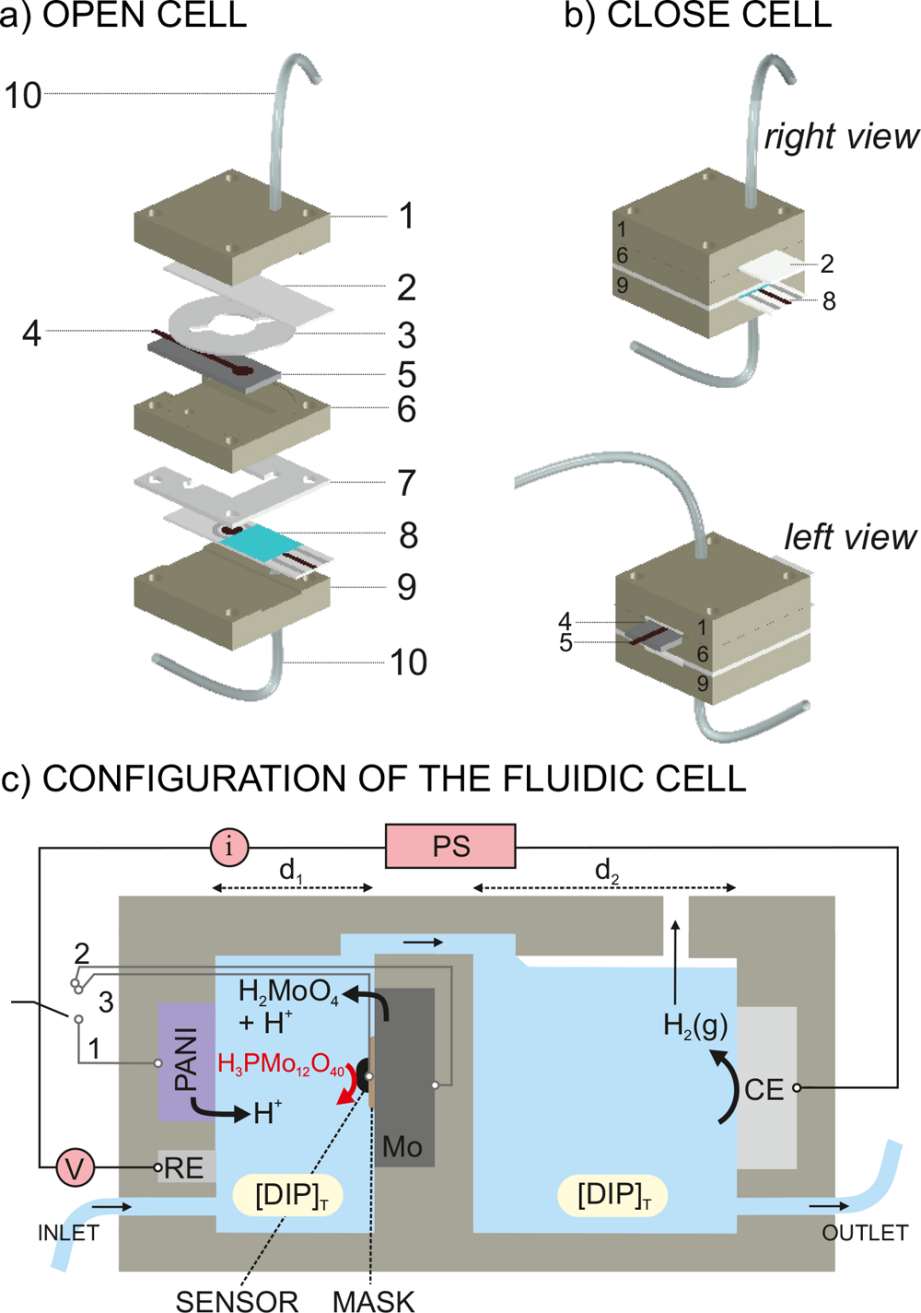
(a) Elements of the microfluidic cell. (1) Top electrode holder; (2) Electrode 1 (DRP-150 modified with PANI); (3) 0.50 mm thick silicon rubber spacer; (4) the screen-printed carbon electrode in Electrode 2; (5) the Mo electrode in Electrode 2; (6) a middle holder for electrode 2; (7) a 1 mm thick silicon rubber spacer; (8) Electrode 3 (an unmodified DRP-150); (9) the bottom electrode holder; and (10) tubings. (b) Closed cell viewed from the right and left. (c) Schematic of the flow cell configuration. [DIP]_T_ = total dissolved inorganic phosphorous; PANI = polyaniline; PS = power supply; RE = reference electrode; CE = counter electrode; Mo = molybdate electrode; V = voltage; I = current; g = gas; 1, 2, and 3 refer to the working electrodes used in steps 1 (acidification), 2 (molybdenization), and 3 (DIP sensing); d_1_ and d_2_ are the thicknesses of the two reservoirs (500 and 1000 μm, respectively).

Although significant progress has been made toward achieving decentralized DIP measurements, there is still a need for a portable and reagentless DIP detection method, preferably capable of working at soft operational conditions to avoid the risk of unnecessary sample alteration, and that can be integrated into submersible and reusable probes for automatic and continuous monitoring.^10,17^ In this study, we present an all-in-one electroanalytical concept capable of forming and measuring the phosphomolybdate complex in seawater with demonstrable accuracy. It involves an actuator-sensor system, as illustrated in Figure 1, in which a series of electrodes are installed for the (i) H^+^ delivery from polyaniline (PANI) activated at a mild potential (actuator 1),^20^ (ii) combined H^+^ and MoO_4_^2–^ delivery from a Mo electrode activated at a mild current (actuator 2), and (iii) H_3_PMo_12_O_40_ electrochemistry (cyclic voltammetry, CV, and SWV) for indirect DIP quantification. Such (electro)chemical conditions are created in a fluidic cell (Figure 2a) that confines the sample to a very thin domain in which the mass transport effect is greatly minimized, consequently keeping sample modification and reaction times to a minimum. The principles behind the sensor and the device are thoroughly investigated, and the analytical significance is demonstrated with validated measurements in five water samples from the Baltic Sea.

## Experimental Section

Voltammetry experiments were performed with an Autolab PGSTAT204 potentiostat running on Nova 2.1 (Metrohm Nordic AB, Sweden). We used the following electrodes purchased from Metrohm Nordic AB (Sweden): screen-printed carbon electrode (DRP-150), glassy carbon working electrode (GCE; diameter of 5 mm, RDE.GC50.S), Pt electrode (6.0331.010), and a Ag/AgCl single-junction reference electrode (EQCM. refEL.S). A Mo plate was also used as an electrode (Sigma-Aldrich, 357200-25.6G). Electrochemical measurements in “beaker configuration” were performed using a three-electrode system with the GCE as the working electrode (WE), the Ag/AgCl single-junction reference electrode (RE), and the Pt rod as the counter electrode (CE). For the experiments conducted in the microfluidic cell (Figure 2a), three types of electrodes were prepared and/or utilized. Electrode 1 was the DRP-150 with a carbon-based WE modified with a film of electropolymerized PANI (150 CV scans in 0.1 M aniline/0.5 M H_2_SO_4_). Electrode 2 was the Mo plate modified with a custom-made screen-printed carbon electrode (SPCE). Electrode 3 was an unmodified DRP-150. More details about the preparation of the electrodes are presented in the Supporting Information. Figure S1 presents a real picture of the device.

The fluidic cell was designed in AutoCAD 2020 and printed with an Ultimaker 3 3D printer (Ultimaker B.V., the Netherlands) using PLA filament (Ultimaker, the Netherlands). The rubber spacers (thicknesses of 0.5 and 1 mm) were purchased from (Ecoflex, USA) and cut to an appropriate size to create the microfluids for the cell. The cell is composed of two internal compartments: the first compartment is situated between Electrode 1 and Electrode 2, while the second is situated between Electrode 2 and Electrode 3. The WEs of actuator 1 and actuator 2, the sensor, and (a common) RE are placed in the first compartment, while the common CE (the carbon part of the DRP-150; Electrode 3) is placed in the second compartment. The Ag element in the DRP-150 in Electrode 1 acts as the common RE. The PANI-C element of Electrode 1 acts as the WE of actuator 1 and acidifies the sample upon the application of 0.4 V for 300 s. The Mo element in Electrode 2 acts as the WE of actuator 2 and delivers MoO_4_^2–^ and H^+^ by applying a current of 0.15 mA for 300 s. Finally, the C path in Electrode 2 acts as the WE of a DIP sensor that uses either CV or SWV. The potential was switched in the cathodic direction to ensure the partial reduction of Mo(VI) centers in the phosphomolybdate complex to Mo(V).

## Results and Discussion

### Concept

Here, we present an all-in-one electroanalytical methodology for the measurement of DIP concentrations in environmental water samples. The overall principle is translated into a compact portable device that can either be implemented in submersible probes for in situ detection or integrated into on-site methodologies that require DIP monitoring, such as water remediation studies.^21^ The mechanisms that underlie this approach are based on three electrochemical events that occur in series (Figure 1).

Step 1, termed “acidification”, results in a final pH of around 2.4 in a sample confined in a 500 μm thick reservoir (50 μL). Specifically, the electrochemical properties of PANI are exploited to release a massive number of protons upon the application of a mild anodic potential (0.4 V for 300 s).^20,22^ The acidification process has a threefold function: (i) it displaces the equilibrium of the phosphate species, encouraging the formation of H_3_PO_4_; (ii) it facilitates the electroactivity of the Mo electrode (Figure S2), allowing the oxidation process in Step 2 to form molybdate (VI);^23^ and (iii) it creates the acidic conditions necessary for the formation of the phosphomolybdate complex (with the anion core exhibiting a Keggin structure)^4^ from the orthophosphate present in the sample and molybdate, consequently enhancing the sensing capabilities of Step 3.

Step 2, termed “molybdenization”, involves the combined release of H^+^ and molybdate by the galvanostatic oxidation (0.15 mA for 300 s) of the solid Mo electrode. The formation of the phosphomolybdate complex occurs in a quantitative manner, as Mo(VI) species will be in excess with respect to phosphate species. Step 2 generates additional protons that lead to conditions in which the resultant phosphomolybdate complex is highly stable (pH ∼ 2.0). Then, the derivatization process enables Step 3: the indirect detection of DIP, given that the phosphomolybdate complex is electroactive. Thus, Step 3 involves the electroanalytical detection of phosphomolybdate by partially reducing the Mo(VI) centers to Mo(V) via CV or SWV on the carbon electrode.

### Microfluidic Cell

This section discusses the setup and configuration of the electrodes that are required for Steps 1–3. In essence, we propose an all-in-one device composed of two actuators (for acidification and molybdenization) and a sensor. The corresponding working electrodes (WE_1_, WE_2_, and WE_3_) share a common RE (Ag/AgCl) and a common CE (carbon with a large surface area). Figure 2 describes the microfluidic cell configuration. The final arrangement (Figure 2a) is the result of a thorough investigation into the experimental design that was conducted by assessing the performance of both the individual and combined steps. The two actuators and the sensor are distributed as a series of electrodes as follows: Electrode 1 contains a PANI-based WE_1_ for acidification and the common RE. Electrode 2 is composed of a Mo plate (WE_2_ for molybdenization) and a manually printed carbon path (WE_3_ for DIP sensing). Electrode 3 is the common CE. The fluidic cell contains two chambers that are connected through a micro-channel, with an inlet and an outlet that allow the cell to be filled with the sample through the use of a syringe or a peristaltic pump. Electrodes 1 and 2 are located in the first chamber, while Electrode 3 is located in the other one. Electrical connections are made on both sides of the cell to maximize the available space (Electrodes 1 and 3 on one side, and Electrode 2 on the other side; Figure 2b). The three WEs were sequentially operated using an electronic switch, as shown in the circuitry presented in Figure 2c.

In preliminary designs, we opted for a cell composed of a single chamber that contained all of the electrodes; however, in Step 2, molecular hydrogen was found to be produced at the CE due to water electrolysis. In such a tiny compartment, the hydrogen bubbles were preferentially retained at the surfaces of WE_2_ and WE_3_, limiting the electrochemical efficiency of the cell. Also, the final pH of the sample could be altered, according to eq 3.
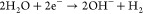
3

Thus, we designed the two-chamber cell to eliminate the effect of hydrogen formation on the output signal and the final sample pH. The compartment that exclusively holds the CE is an open chamber with a thickness of 1 mm (d_2_, Figure 2c). In contrast, the other compartment was designed with a thickness of 500 μm (d_1_) and contains the sample inlet, WE_1_–WE_3_, and RE. The proposed thickness allows the cell to operate in the regime of quasi-thin-layer electrochemistry. Then, WE_1_ and WE_2_ were placed one in front of each other to maximize the area that they could occupy within the available space in the cell, which in turn maximizes their ability to deliver protons and molybdate. While each electrode can be accompanied by either the RE or WE_3_, the incorporation of WE_3_ was only feasible at the surface of the Mo plate (WE_2_). WE_3_ was thus placed on top of WE_2_ using a nonconducting mask that ensured that there was no electrical connection between both (see the Supporting Information). The surface area of WE_3_ was enough to guarantee an acceptable voltammetric measurement of the phosphomolybdate complex.

### Formation and Electrochemical Measurement of the Phosphomolybdate Complex

Recent studies by the Compton group defined the dependency of the speciation of molybdate anion form on pH conditions, based on eqs 4–6.^11^
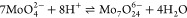
4
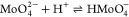
5
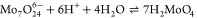
6

At environmental pH (pH > 6), molybdate is mainly found as MoO_4_^2–^. At slightly acidic pH values (3 < pH < 5), the heptameric (Mo_7_O_24_^6–^) or partially protonated (HMoO_4_^–^) forms of molybdate are predominant, as shown by eqs 4 and 5. Specifically, when the pH is between 3 and 5 and the molybdate has an initial concentration of 8 mM MoO_4_^2–^, the predominant molybdate form is described by eq 4, while an initial concentration of 1 mM MoO_4_^2–^ results in the product described by eq 5.^11^ Finally, H_2_MoO_4_ is the primary species when pH is lower than 3 (eq 6). Thus, this latter form is the compound involved in the formation of the phosphomolybdate complex traditionally detected in DIP analyses (eq 1). Importantly, an excess of molybdate can ensure the formation of phosphomolybdate ions by shifting the equilibrium described in eq 1 toward the products.^11^

The phosphomolybdate complex is known to exhibit two pairs of characteristic redox peaks at pH ≤ 2, and the associated electrochemistry appears to be governed by its adsorption onto the electrode surface.^11,15−17^ Overall, different Mo units are converted between the +6 and +5 redox states, as shown in eqs 7 and 8.^17^
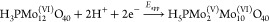
7
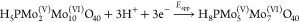
8

**Figure 3:**
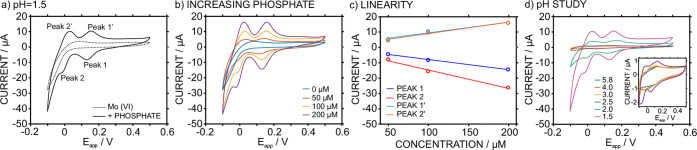
(a) CVs observed in 1.14 mM H_24_Mo_7_N_6_O_24_/0.1 M NaCl (pH = 1.5) before and after the addition of 100 μM phosphate. (b) CVs at increasing phosphate concentrations in 1.14 mM H_24_Mo_7_N_6_O_24_/0.1 M NaCl (pH = 1.5) background solution. (c) Corresponding calibration curves based on the absolute peak currents of the four appearing waves. (d) CVs observed in solutions of 100 μM phosphate/1.14 mM H_24_Mo_7_N_6_O_24_/0.1 M NaCl at increasing pH. The applied potential was scanned from 0.5 to −0.1 V and back. Scan rate = 50 mV s^–1^.

In this paper, a relatively high concentration of phosphate (200 μM) was used to conduct experiments with well-defined redox peaks and clearly identify the electrochemistry of Mo(VI). Following this, a ca. 1:40 P/Mo(VI) molar ratio was generated using 1.14 mM ammonium molybdate tetrahydrate; eq 4 was used to calculate the concentration of Mo(VI). This was used to guarantee the formation of 12-heteropoly phosphomolybdate ions (the Keggin ions).^4^Figure 3a presents the voltammogram of a sample processed using the developed fluidic cell (only the detection part with WE_3_, RE, and CE shown in Figure 2) at pH 1.5, which is adequate for the formation of the phosphomolybdate complex, in 0.1 M NaCl solution. The potential was scanned from −0.1 to 0.5 V (and back to −0.1 V) at 50 mV s^–1^.

Two cathodic peaks (peak 1 at 0.13 V and peak 2 at −0.02 V) and their corresponding anodic parts (peak 1′ at 0.15 V and peak 2′ at 0.03 V) were observed. The cathodic peaks were assigned to eqs 7 and 8, respectively, in accordance with the literature.^17^ In contrast, the voltammogram observed at analogous conditions but in the absence of phosphate did not reveal the aforementioned peaks; instead, there was an increase in the reduction current between 0 and −0.1 V as well as the appearance of an oxidation peak at 0.07 V (peak current of 2.65 μA). Thus, peaks 1/1′ and peaks 2/2′ are caused by the presence of the phosphomolybdate complex. A control experiment in the absence of molybdate, with and without phosphate, using the same electrolyte and pH conditions, revealed purely capacitive behavior (Figure S3); this is consistent with our hypothesis. Furthermore, when the voltammograms were obtained at increasing concentrations of phosphate in the solution, increased peak currents were detected (Figure 3b).

Accordingly, linearity for each of the four peaks (absolute current output, i) with respect to the phosphate concentration (equivalent to DIP) in the sample was investigated. The fitted equations are presented in eqs 9–12.
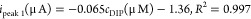
9
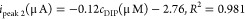
10
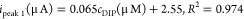
11
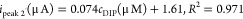
12

In principle, the most suitable peak based on its relationship with the DIP was peak 2, which exhibited a good balance between sensitivity and linearity. However, the signal of peak 1 was more reproducible, while the current of peak 2 was found to decrease slightly over five consecutive CV scans (e.g., at 100 μM of phosphate, peak 1 and peak 2 had values of −8.19 ± 0.053 μA and −16.57 ± 0.221 μA, respectively). Previous works suggested that peak 2 is usually less reproducible than peak 1 because of the adsorption of phosphomolybdate ions on the surface of the working electrode.^14^ In general, the results suggested that it was possible to detect the phosphomolybdate complex, which is equivalent to DIP, by monitoring the reduction peak at 0.13 V.

Next, experiments were conducted at varying pH conditions (from 1.5 to 5.8) while keeping the Mo(VI) and phosphate concentrations constant in the solution. The corresponding voltammograms are presented in Figure 3d. The absolute current of each peak tended to decrease with increasing pH; in some cases, the peak even disappeared. While the optimal peak currents occur at pH 1.5, a pH of ∼2.0 may also be suitable for DIP detection as long as the sensitivity in the concentration range required for the analytical application is acceptable. Above pH 3, there was no clear evidence of any redox behavior at the selected experimental conditions. This is likely due to the difficulty of forming the P-Mo complex.

Overall, the electrochemical readout under controlled chemical conditions in the microfluidic cell (i.e., manually fixing the pH and Mo(VI) concentrations) was analogous to the results of beaker-based experiments (Figure S4). However, the potentials of the four voltammetric peaks shifted slightly to more positive values (peak 1 at 0.23 V, peak 2 at 0.12 V, peak 1′ at 0.30 V, and peak 2′ at 0.19 V) due to the change in electrodes (the GCE as the WE and a single-junction Ag/AgCl as the RE). The results also indicated an increase in the peak current with phosphate concentration in the solution (Figure S4b) and, between a pH range of 1.5 to 5.8, peaks 1 and 2 were only distinguishable at pH < 3.

### Actuator 1: Electrochemically Controlled Acidification Using PANI Film

Having demonstrated the electrochemical detection of the phosphomolybdate complex in the developed microfluidic cell, the possibility of lowering the natural pH of the environmental sample to the required levels for the formation of the P-Mo complex via actuator 1 was investigated. It is here anticipated that this purpose is indeed achieved when the acidification resulting from the PANI and Mo materials are combined (i.e., actuator 1 + actuator 2). Advantageously, this concept does not require the addition of an external reagent, nor does it require the use of harsh electrochemical conditions to acidify the sample, in contrast to previously reported approaches (see the Introduction section). To monitor the pH change of the sample, the microfluidic cell was slightly modified and a potentiometric pH sensor was installed to replace either the Mo plate or the PANI electrode (Figure S5).

**Figure 4:**
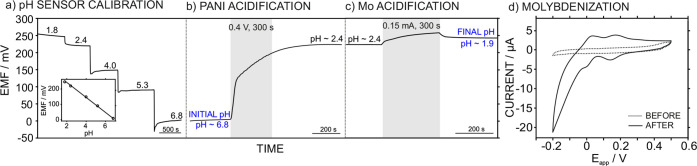
(a) Dynamic EMF response of the pH sensor calibrated from pH 1.8–6.8 in the microfluidic cell. Inset: corresponding calibration graph. (b) EMF profile of the pH sensor during the treatment of a seawater sample with PANI-based acidification. (c) EMF profile of the pH sensor during the treatment of a seawater sample (pH modified down to 2.4) with Mo-based acidification. (d) CV response of a seawater sample (pH modified down to 2.4) before and after the molybdenization process. The applied potential was scanned from 0.5 to −0.1 and back. Scan rate = 50 mV s^–1^.

Figure 4a–c illustrates the calibration of the pH sensor in the cell (Figure 4a) and the electromotive force (EMF) profiles observed when a seawater sample (initial pH of ∼6.8) was treated with actuator 1 (Figure 4b) followed by actuator 2 (Figure 4c). It should be noted that in the experiment with only actuator 2, the pH achieved with actuator 1 was mimicked by manually acidifying a new plug of the seawater sample. Thus, the acidification experiment was conducted by placing the seawater sample (pH = 6.8) into the microfluidic cell, stopping the flow, and applying 0.4 V against the open-circuit potential (OCP) for 300 s to the WE_1_ (PANI-based electrode). The dynamic EMF revealed an initial constant value (coinciding with the initial pH of the sample), before gradually increasing, which coincided with the polarization of WE_1_. Finally, a constant potential was established, corresponding to the final pH achieved in the sample due to the release of protons from PANI. The final value of pH∼2.4 was consistent with previous results reported by our group.^20,22,24^ Then, a new sample plug was introduced, this time at pH 2.4. Once molybdenization occurs in actuator 2 (0.15 mA for 300 s), the final pH of the seawater was 1.9, which was low enough to form the desired phosphomolybdate complex. When three seawater samples with initial pH ranging from 6.8 to 7.0 were acidified, the final pH obtained in average was 1.99 ± 0.05, confirming the validity of the acidification concept.

### Actuator 2: Electrochemically Controlled Molybdenization from the Mo Plate

The delivery of the Mo(VI) species needed to form the phosphomolybdate complex was investigated using a Mo plate (WE_2_), according to eq 2. First, the dynamic current provided by the Mo electrode was recorded as the potential was adjusted between 0 and 0.2 V (50 mV s^–1^) in a 0.1 M NaCl solution at both acidic and neutral conditions (Figure S6a). Further potentials were not investigated to limit the utilized settings to mild conditions.

In such circumstances, the expected species to be formed from the Mo plate should be HMoO_4_^–^, based on the potential-pH diagram of the Mo–H_2_O system (Figure S6b), which should manifest as a current increment. However, from approximately 0.02 to 0.12 V, the formation of a passive surface of oxides was evidenced (constant current of 0 mA was shown). While the trend observed for the current was very similar at both tested pHs, acidic conditions appeared to favor higher currents between 0.12 and 0.2 V. In any case, it was realized that electrode reusability was not convenient since surface modification, likely due to the insolubility of the passive oxides, appeared.

Instead, the use of a constant current to oxidize the Mo plate was investigated. The applied current was varied between 0.15 and 1.5 mA, and the dynamic potentials were registered in 0.1 M NaCl solution at pH 2.5 for 300 s (Figure S6c). In general, the higher the applied current, the higher the observed potential, with the values being rather mild. In addition, it was found that the use of the Mo plate for subsequent molybdenization processes caused the electrode to be gradually covered with a film that changed its electrochemical properties; this effect has been previously reported in other studies.^23^ The undesired film was more evident at higher applied currents, and therefore, the optimal current that would minimize the requirements for electrode restoration (i.e., after ca. 10 measurements) was determined to be 0.15 mA for 300 s. Furthermore, this condition ensures the delivery of an excess of Mo(VI) with respect to the DIP expected in the water sample, as described below.

Considering the change in pH detected during the molybdenization (i.e., from 2.4 to 1.9), the number of moles of released protons can be calculated; consequently, the released Mo(VI) can be estimated via stoichiometry from eq 2 (see the Supporting Information for the entire calculation process). Thus, considering an internal volume for the cell of 50 μL, the Mo(VI) delivered by the molybdenization process has an approximate molar ratio of 1:54 P/Mo(VI) in a sample containing 20 μM DIP (a relatively high phosphate value for ocean surface porewater).^19,25^ This situation should be sufficient to generate the phosphomolybdate complex, which was confirmed with voltammograms from a 20 μM phosphate solution at a fixed pH of 2.4 (the pH achieved with PANI-based acidification) before and after molybdenization (Figure 4d). Effectively, peaks associated with the phosphomolybdate complex only appeared after molybdenization.

### Investigation of the All-in-One Electrochemical Actuator-Sensor System for Voltammetric DIP Detection

**Figure 5:**
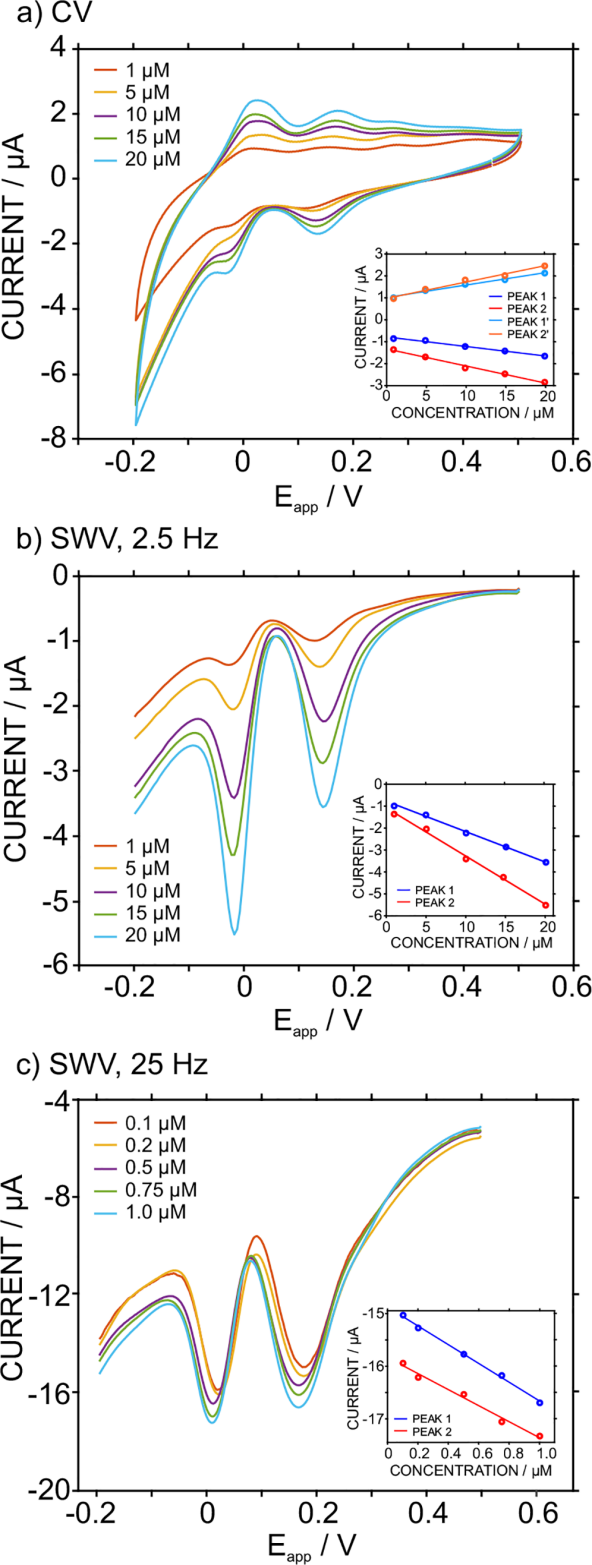
(a) CV response for phosphate concentrations between 1 and 20 μM. Scan rate = 50 mV s^–1^. (b) SWV responses for phosphate concentrations between 1 and 20 μM. Frequency = 2.5 Hz. (c) SWV responses for phosphate concentrations between 0.1 and 1.0 μM. Frequency = 25 Hz. Insets: corresponding calibration graphs. Background: 0.1 M NaCl, initial pH of 6.8.

The detection of phosphate was then performed using the complete actuator-sensor system: Step 1 [H^+^ release from PANI, 0.4 V, 300 s], Step 2 [molybdate and H^+^ release from the Mo plate, 0.15 mA, 300 s], and Step 3 [CV or SWV]. Figure 5a shows the CVs (from 0.5 to −0.2 V and back, 50 mV s^–1^) obtained at increasing phosphate concentrations (1–20 μM, 0.1 M NaCl background at pH = 6.8) in the sample solution, while Figure 5b presents the voltammograms collected by SWV detection (frequency of 2.5 Hz, modulation amplitude of 25 mV, and potential step of 5 mV). In both cases, the voltammetric peaks increased as the phosphate concentration increased and displayed an acceptable degree of linearity in the inspected phosphate concentration range (eqs 13–18).
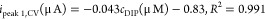
13
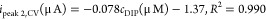
14
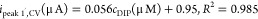
15
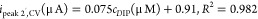
16
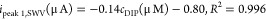
17
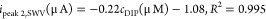
18

While, in principle, any peak could be used to create a calibration graph with an analytical meaning, peak 2 appears to provide better sensitivity. However, it was found that slight variations in the final pH achieved in the sample (i.e., after Steps 1 and 2) more strongly affected the shape of peaks 2/2′ than peaks 1/1′ (for example, see the CVs for pHs 1.5, 2, and 2.5 in Figure S4). Hence, peak 1 was selected for further analytical investigation.

The detection of phosphate concentrations lower than 1 μM was only plausible with SWV since this technique compensates for capacitive current contribution, resulting in a superior signal-to-noise ratio compared to CV.^11,17^ Indeed, the use of higher frequencies results in more well-defined voltammetric peaks (see the calibration graphs in Figure S7), reducing the LOD in turn. Higher modulation amplitudes also enhanced the current of the voltammetric peaks (Figure S8).

Figure 5c presents the results of using the developed actuator-sensing concept with SWV at 25 Hz, a modulation amplitude of 50 mV, a potential step of 2 mV, and a scan rate of 50 mV s^–1^ in phosphate concentrations ranging from 0.1 to 1 μM. The absolute currents of both peaks were found to linearly increase with phosphate concentration (eqs 19 and 20). Once again, peak 1 was confirmed to be the best as the analytical signal.
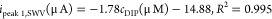
19
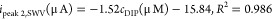
20

The need to change the frequency of the SWV experiment was further investigated with calibration graphs ranging between 0.1 and 20 μM at 2.5 and 25 Hz (Figure S9). In essence, higher frequencies (25 Hz) allowed for better discrimination between the lower concentrations (0.1–1 μM), while lower frequencies allowed for better discrimination between higher concentrations (1–10 μM). Consequently, the appropriate calibration graph should be used depending on the phosphate concentration expected in the sample to maximize the reliability of the analysis.

### Investigation of the Potential Interference of Silicate

Silicate concentrations in seawater and porewater generally vary from <0.1 to 150 μM, with these increasing with the depth of the water column.^26^ When analyzing the phosphate (or DIP) concentration in natural water samples based on the formation of the phosphomolybdate complex with Keggin structure, orthosilicate (silicate) is a potential interferent because it may form analogous electroactive complexes with Mo(VI).^16^ Under the chemical conditions needed for phosphomolybdate complex formation, the silicomolybdate complex SiMo_12_O_40_ may also be generated. Nevertheless, the process (and its kinetics) is known to strongly depend on the H^+^/Mo(VI) molar ratio as well as the presence of other substances capable of forming molybdate-based complexes (e.g., phosphate and antimony).^27^ Thus, decreasing the pH facilitates the generation of polysilicon acids, avoiding the formation of silicomolybdate complexes in favor of phosphomolybdate complexes.

**Figure 6:**
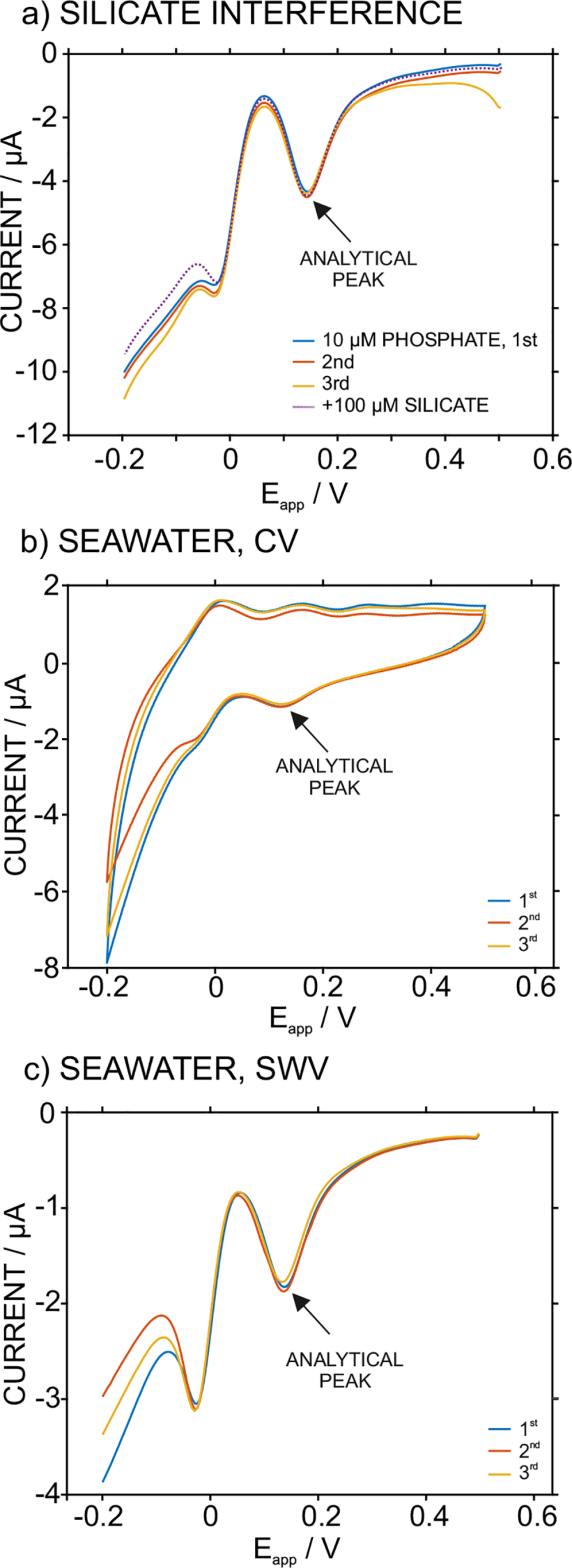
(a) Study of silicon interference in the actuator-sensor cell. Frequency of 2.5 Hz, amplitude of 25 mV, and a potential step of 5 mV. (b) Triplicate CV signals for sample #2. Scan rate of 50 mV s^–1^. (c) Triplicate SWV signals for sample #2. Frequency of 2.5 Hz, an amplitude of 25 mV, and a potential step of 5 mV.

To investigate the influence of silicates on the response of the actuator-sensor cell, we performed an experiment in which the SWV signal of 10 μM phosphate concentration was obtained in triplicate and then, the addition of 100 μM silicate concentration was accomplished. The results are presented in Figure 6a. Notably, a phosphate concentration of 10 μM was selected because it reflects the concentrations that are expected in the seawater samples that will be analyzed in this work. Then, the triplicate measurements of the 10 μM phosphate solution will serve to identify whether any variation detected because of the presence of silicate in the sample is not originated from the intrinsic variation of the sensor response. The average current found for the analytical peak was −4.36 ± 0.08 μA, which represents a variation of 1.8%. The variation for the peak current when silicate was present in the sample was 1.2% (peak current of −4.41 μA), which is negligible and lower than the intrinsic variation observed for the methodology reproducibility.

### Detection of DIP in Seawater Samples

Five pore-seawater samples from the Baltic Sea were analyzed in triplicate by the developed methodology as well as by ion chromatography (IC). After calibrating the system using standards containing increasing phosphate concentrations in 0.1 M NaCl solution with initial pH of 6.8, the samples were injected into the cell without any prior treatment. Steps 1 and 2 were sequentially applied to both the standards and samples before being analyzed via CV or SWV (Step 3). In the latter technique, a frequency of 2.5 Hz and a modulation amplitude of 25 mV were used since the expected phosphate (or DIP) concentration was >1 μM. Figure 6b,c shows the results obtained for one of the seawater samples (Sample #2) analyzed by CV and SWV, while the signals from the entire pool of samples are presented in Figure S10. The reproducibility of the absolute current of the peak selected for analysis was acceptable (6.80 ± 0.92 and 7.54 ± 0.67 μA for CV and SWV, respectively, in Sample #2).

Table 1 collects all of the results and calculates the differences between the DIP concentrations obtained with either CV or SWV as well as those estimated with IC. The results pointed out an acceptable agreement between the techniques, with percentage differences ranging from 0.5 to 9%, demonstrating that the developed analytical tool for DIP detection had a good degree of accuracy. Slightly better results were observed for SWV (i.e., lower percentage differences) than CV. Furthermore, values for the *t*-score for CV (*t* = 0.82) and SWV (*t* = 2.52) were calculated to be lower than the critical threshold (*t* = 2.77, *p* = 95%), indicating that they were no statistically significant differences between the CV and SWV and the IC.

**Table 1:** DIP Analysis in Seawater Samples

	DIP, μM			diff with IC, %	
sample	CV^a^	SWV^a^	IC^b^	CV	SWV
1	10.52 ± 0.34^c^	10.81 ± 0.58	11.5	8.5	6.2
2	6.80 ± 0.92	7.54 ± 0.67	7.5	8.7	0.5
3	13.94 ± 0.31	11.72 ± 0.28^c^	12.5	8.9	8.2
4	14.16 ± 3.46	14.52 ± 0.67	15.8	8.7	6.4
5	7.23 ± 2.29	6.80 ± 1.20	7.0	3.8	2.8

a Average ± standard deviation (*n* = 3).

b The variation of IC measurements was calculated to be ± 4.5%.

c *n* = 2.

## Conclusions

The accurate detection of DIP in seawater samples is possible using an actuator-sensor system that provides all-in-one H^+^ and Mo(VI) delivery to allow for the electrochemical detection of the phosphomolybdate complex. The cell is designed to provide all of the needed electrodes for a series of electrochemically driven steps: acidification, molybdenization, and detection. Chemical conditions for the formation of the phosphomolybdate complex are achieved via the combined acidification from PANI and solid Mo materials, which achieves a final sample pH of ∼2.0 while guaranteeing the sufficient delivery of Mo(VI) to achieve a molar ratio in excess of 1:54 P/Mo(VI). An acceptable degree of linearity between DIP concentration and the peak current in the voltammograms was found in the phosphate concentration ranges between 1 and 20 for CV and 0.1 and 20 μM for SWV. Validated DIP measurements in five seawater samples confirmed the adequate accuracy of the analytical methodology. In principle, the concept should be suitable also for the analysis of river and lake samples (i.e., lower salinity), which is something to be demonstrated. The significance of the results paves the way for the development of a new generation of all-in-one devices, which addresses the current limitations in the in situ monitoring of nutrients (i.e., selectivity, LOD, portability, etc.) in aquatic systems. This concept is expected to allow for real-time data with unprecedented spatial and temporal resolutions, the provision of information connected to anthropogenic nutrient discharges, and the improved monitoring of seawater restoration actions.

## Supporting Information Available

Materials and instruments; protocols and additional calculations; optimization of the Mo polarization; additional control experiments; experiments in the beaker; cells for pH monitoring in the sample; optimization of the SWV conditions; and signals of the seawater samples (PDF)
