# Supplementary material for: Portable All-in-One Electrochemical Actuator-Sensor System for the Detection of Dissolved Inorganic Phosphorus in Seawater
Source: Anal Chem. 2023 Feb 1;95(8):4180–9. doi: 10.1021/acs.analchem.2c05307 (PMC9979141; doi:10.1021/acs.analchem.2c05307)
Supplement: Supplementary file 1 — ac2c05307_si_001.pdf [file ac2c05307_si_001.pdf]

Supporting Information for:

Portable All-in-one Electrochemical Actuator-Sensor System for the Detection of Dissolved Inorganic Phosphorus in Seawater

Chen Chen[a], Alexander Wiorek[a], Alicia Gomis-Berenguer[a], Gaston A. Crespo[a,b], and Maria Cuartero[a,b,*]

[a]*Department of Chemistry, School of Engineering Science in Chemistry, Biochemistry and Health, Royal Institute of Technology, KTH, SE-100 44 Stockholm, Sweden*

[b]*UCAM-SENS, Universidad Católica San Antonio de Murcia, UCAM HiTech, Avda. Andres Hernandez Ros 1, 30107, Murcia, Spain*

Corresponding author (*): mariacb@kth.se

Table of Contents

1. **Experimental Section**........2

Chemicals, materials and instrumentation........2

Protocols for the preparation of the electrodes.........2

2. **Calculations**........3

3. **Figures**........4

Figure S1........4

Figure S2........5

Figure S3........5

Figure S4........6

Figure S5........7

Figure S6........8

Figure S7........9

Figure S8… 9

Figure S9........10

Figure S10........11

4. **References**........12

1. Experimental Section

Chemicals, materials and instrumentation. All solutions were prepared with doubly deionized water with a resistance of 18.2 MΩ cm^{-1} (Milli-Q water systems, Merck Millipore, USA). Sodium phosphate monobasic ($NaH_2PO_4 \cdot H_2O$), sodium chloride (NaCl), ammonium molybdate tetrahydrate ($H_{24}Mo_7N_6O_{24} \cdot 4H_2O$), sodium hexa-fluorosilicate (F_6Na_2Si), aniline (C_6H_7N, ACS reagent grade), sulfuric acid (H_2SO_4, 95.0 – 97.0 % w/w), sodium hydroxide (NaOH, 1M aqueous solution) and the molybdenum (Mo) electrode were purchased from Sigma-Aldrich. Hydrochloride acid (HCl, regent grade, 1M) was acquired from VWR chemicals. The carbon paste (C2030519P4) was purchased from Henkel, Germany. Polyester sheets (0.075 mm) were purchased from RS components, Sweden. The porewater samples were collected from Stockholm Archipelago, the Baltic Sea, Baggensfjärden (59.319561 N, 18.34675 E) and Farstaviken (59.32628 N, 18.35872 E) (17 m depth). The sediment samples were centrifugated at 2500 rpm for 15 mins and the supernatant seawater was collected for further analysis. Calculations were accomplished in MATLAB_R2020b software. The pH measurements were measured using an EMF16 potentiometric multichannel data acquisition device (Lawson Laboratories, Inc., USA). The seawater samples were analyzed with an ion-chromatography system (850 Professional IC Anion, Metrohm, Switzerland).

Protocols for the preparation of the electrodes. The Mo plate (3 cm × 1 cm × 0.1 cm) was obtained cutting the purchased raw metal foil with a hand lever shear (Peddinghaus 2R-3/300, Germany), being polished with sandpaper (P1200), washed with distilled water and dried in air before usage in the microfluid cell. The carbon screen-printed electrode was prepared on a polyester substrate after being cleaned with ethanol. The conductive carbon ink was screen-printed using stencils (designed in Corel Draw and fabricated by Coated Screens Scandinavian AB, Sweden) on the polyester substrate, and dried in the oven at 100°C for 1 hour, according to a previously published protocol.[1] The diameter of the SPCE was 5 mm and the thickness of the conducting path was 0.02 mm. The PANI-based electrode was prepared via electropolymerization in 0.1 M aniline/0.5 M H_2SO_4 using cyclic voltammetry (CV), as described elsewhere.[2] The PANI-based pH sensor was fabricated on the carbon working electrode in the DRP-150) using 0.1 M aniline/0.5 M H_2SO_4, according to a previously published protocol.[3] After each measurement (steps 1–3), the PANI-based electrode was regenerated in 10 mM H_2SO_4 solution, by applying a constant potential at 0 V vs the reference electrode for 300 s, allowing the electrode to be used for at least two weeks with high efficiency of proton delivery.[2] The Mo plate can be used for at least 10 subsequent measurements (steps 1–3) without any restoring treatment. After 10 measurements, it is convenient to polish it with sandpaper (P1200) and rinse with water to maintain a high efficiency during the molybdate delivery process.

2. Calculations

The amount of Mo(VI) delivered to the sample by molybdenization

The detection compartment inside the fluidic cell has a volume of 50 µL. Considering this volume, the delivered moles of H^+ can be calculated from the pH changed measured by the pH sensor before and after molybdenization. And this can be translated into moles of Mo(VI) considering the chemistry underlying the molybdenization process, as described in **Eq. 2** in the main manuscript. In essence, the following relationships apply, and the corresponding calculations are presented in the table below:

$$[H^+] = 10^{-pH} \quad (S1)$$

$$n_{H^+} = [H^+] \times V_{cell} \quad (S2)$$

$$[Mo_4^{2-}] = \frac{[H^+]}{8} \quad (S3)$$

$$n_{Mo_4^{2-}} = [Mo_4^{2-}] \times V_{cell} \quad (S4)$$

	**Before molybdenization**	**After molybdenization**
pH	2.4	1.9
$[H^+]$ / mM	3.89	12.59
n_{H^+} / mol	1.99×10^{-7}	6.29×10^{-7}
delivered n_{H^+} / mol	–	4.30×10^{-7}
$[MoO_4^{2-}]$ / µM	–	1075
delivered MoO_4^{2-} / mol	–	5.37×10^{-8}

Overall, this calculation indicates that the molybdenization process provides enough molybdate to generate the phosphomolybdate complex needed for DIP analysis in seawater samples.

3. Figures

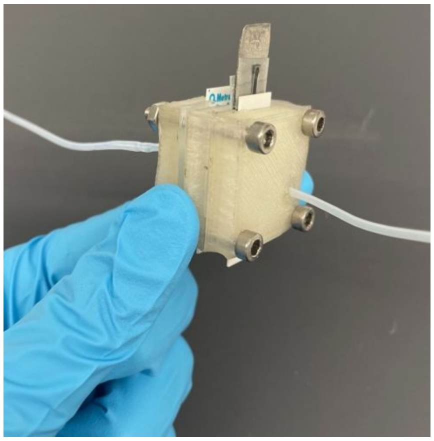

Figure S1. Real picture of the electrochemical cell for DIP detection.

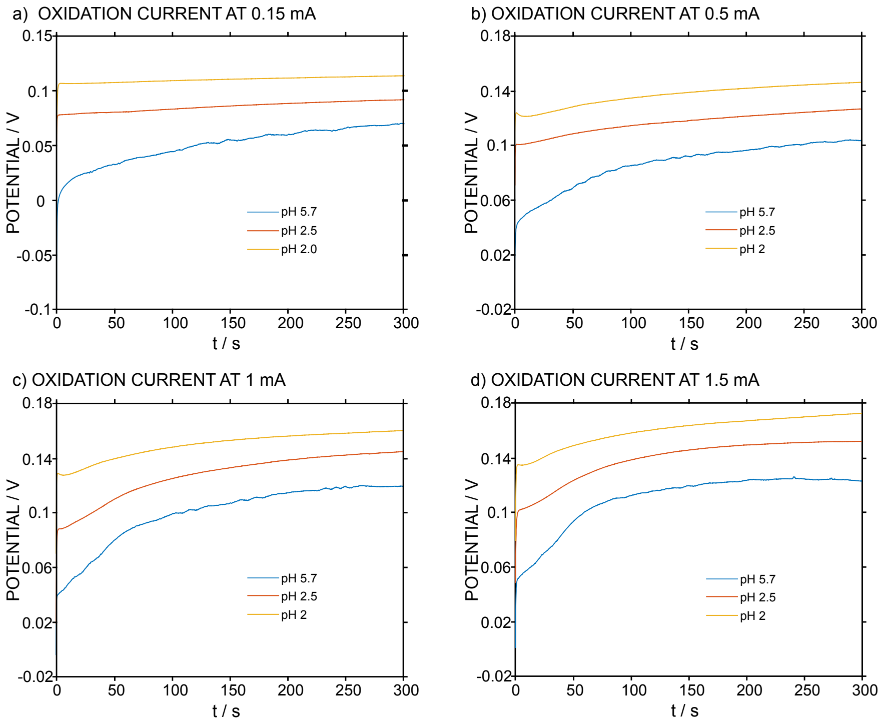

Figure S2. Dynamic potential profiles observed for Mo electrodes polarized at **(a)** 0.15, **(b)** 0.5, **(c)** 1.0 and **(d)** 1.5 mA in 0.1 M NaCl solution at pH values of 2.0, 2.5 and 5.7. Notably, at lower pH conditions, higher potential is reached at the same polarized current, which confirms the release of Mo(VI) during the oxidation process.

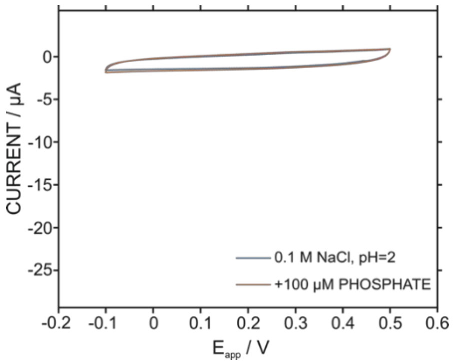

Figure S3. Voltammograms observed in 0.1 M NaCl solution at pH 1.5, before and after the addition of 100 µM phosphate concentration. The applied potential was scanned from 0.5 to –0.1 and back. The scan rate was 50 mV s^{-1}.

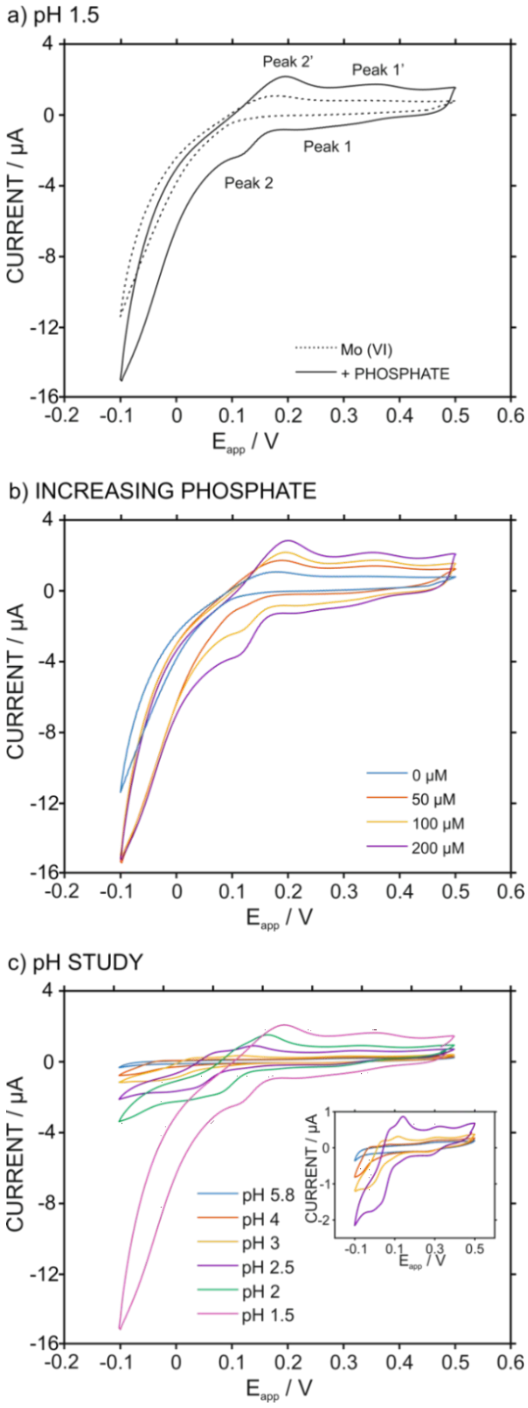

Figure S4. Response of the phosphomolybdate complex in the beaker. **(a)** CVs observed before and after the addition of 100 µM phosphate at pH 1.5. **(b)** CVs at increasing phosphate concentration (0, 50, 100 and 200 µM) at pH 1.5. **(c)** CVs observed in 100 µM phosphate concentration at increasing pH from 1.5 to 5.8. Inset: CVs above pH 2.5. The applied potential was scanned from 0.5 to –0.1 and back. The scan rate was 50 mV s^{-1}. Background: 1.14 mM $H_{24}Mo_7N_6O_{24}$ / 0.1 M NaCl solution.

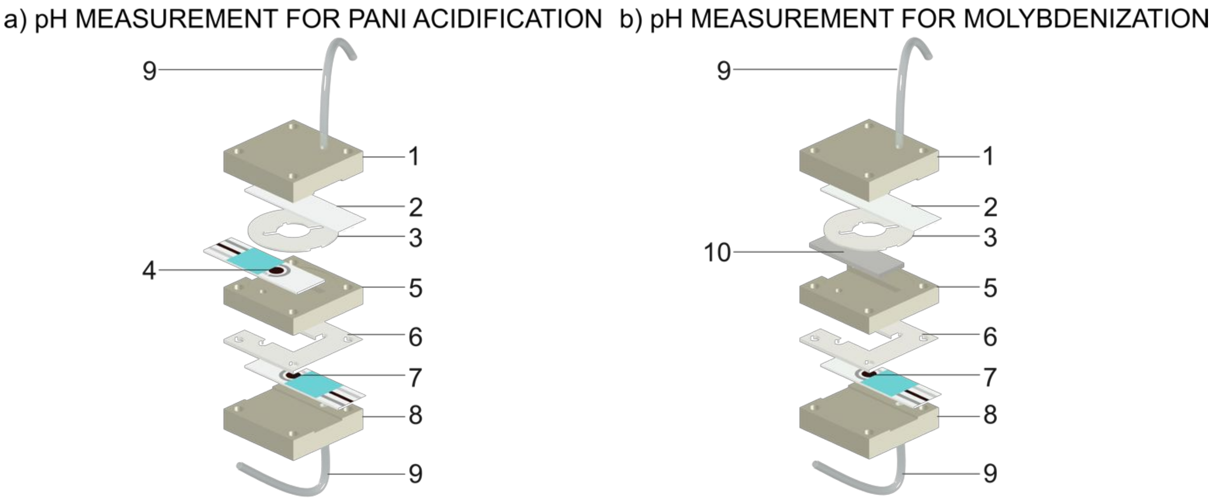

Figure S5. Schemes of the cells including the potentiometric pH sensor for pH monitoring in **(a)** step 1 (PANI-based acidification) and **(b)** step 2 (Mo-based acidification). 1: top electrode holder, 2: PANI-based electrode (DRP-150 modified with PANI), 3: 0.50-mm thick silicon rubber spacer, 4: pH sensor (DRP-150 modified with PANI, 5: a middle holder, 6: 1-mm thick silicon rubber spacer, 7: CE (unmodified DRP-150), 8: bottom electrode holder, 9: tubings, 10: Mo plate.

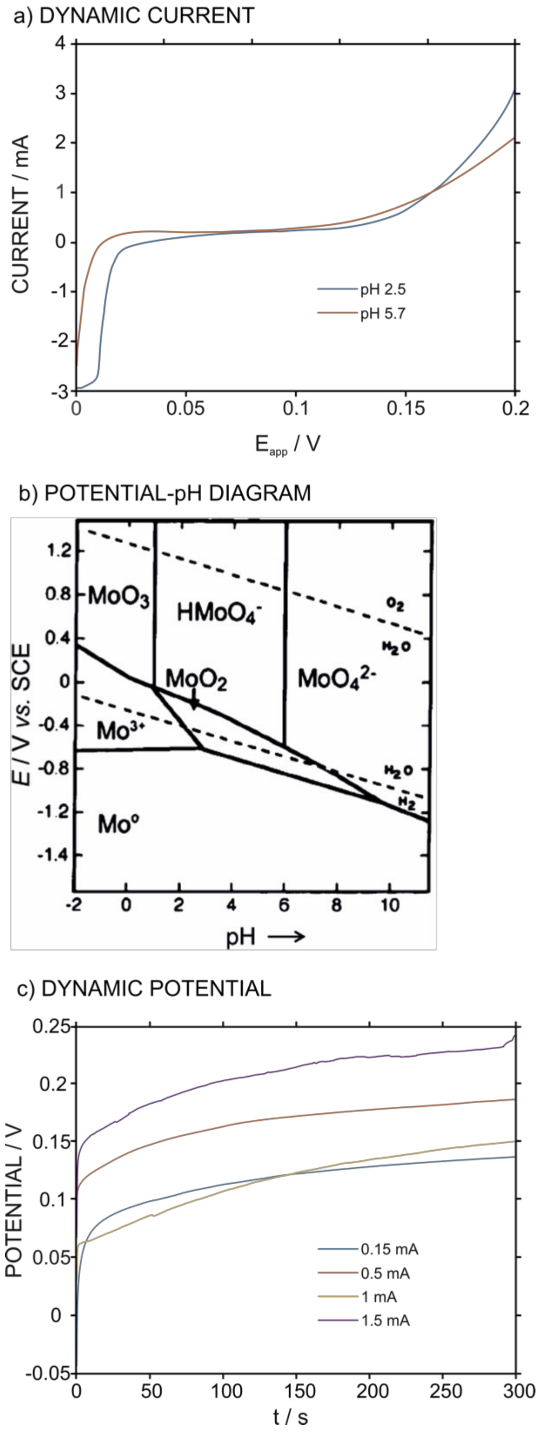

Figure S6. **(a)** Linear sweep voltammogram at a scan rate of 50 mV s^{-1} of the oxidation of molybdenum electrode in cell with NaCl 0.1 M with pH 2.5 and 5.7; **(b)** Potential-pH diagram of a Mo-H_2O system. Reproduced from [4] with permission from John Wiley &Sons, Inc. **(c)** chronopotentiometry for applied currents for the molybdate delivery (pH=2.5). All experiments were performed with 0.1 NaCl as background electrolyte.

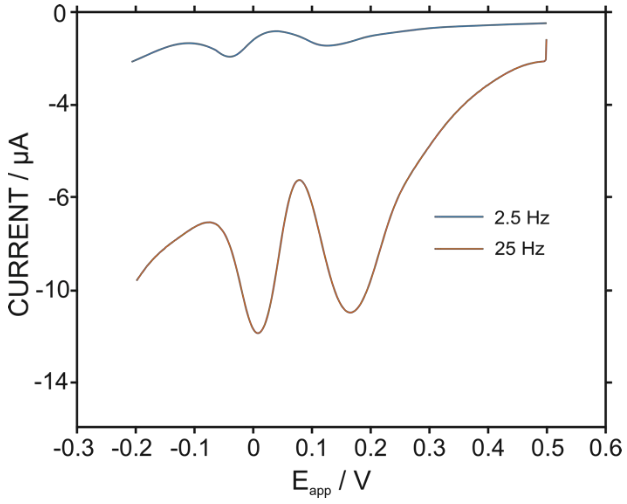

Figure S7. SWV response of 1.14 mM $H_{24}Mo_7N_6O_{24}$ / 1 µM phosphate / 0.1 M NaCl solution at pH 2.0 at different frequencies (2.5 and 25 Hz). Modulation amplitude of 25 mV and potential step of 2 mV.

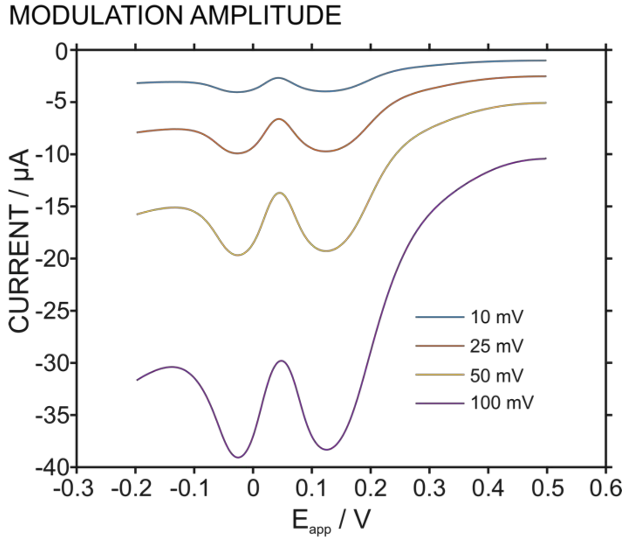

Figure S8. SWV response of 1.14 mM $H_{24}Mo_7N_6O_{24}$ / 10 µM phosphate / 0.1 M NaCl solution at pH 2.0 at different modulation amplitude (10, 25, 50 and 100 mV). The frequency was 25 Hz, and potential step of 2 mV.

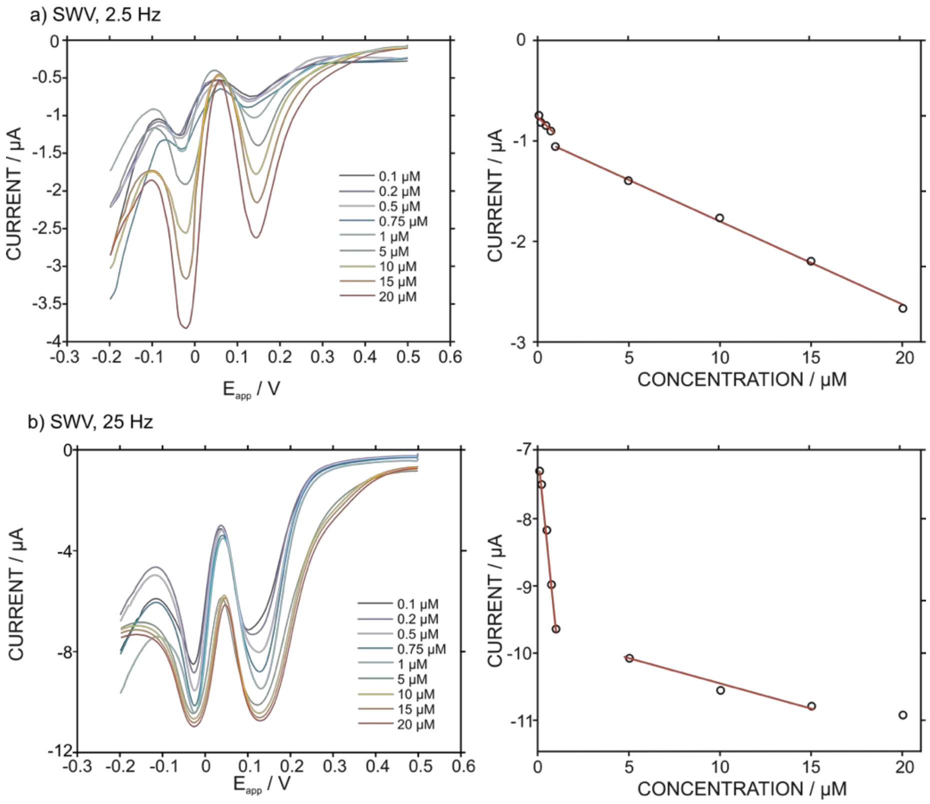

Figure S9. SWVs at increasing phosphate concentrations (from 0.1 to 20 M) with the corresponding calibration graphs (peak 1): **(a)** frequency of 2.5 Hz and **(b)** frequency of 25 Hz. Background: 1.14 mM $H_{24}Mo_7N_6O_{24}$ / 0.1 M NaCl solution at pH 2.0. Modulation amplitude of 25 mV and potential step of 2 mV.

$i_{peak\ 1}(\mu A) = -0.18c_{DIP}(\mu M) - 0.75$, $R^2 = 0.908$, 0.1 – 0.75 µM

$i_{peak\ 1}(\mu A) = -0.084c_{DIP}(\mu M) - 0.96$, $R^2 = 0.998$, 1 – 20 µM

$i_{peak\ 1}(\mu A) = -2.64c_{DIP}(\mu M) - 6.98$, $R^2 = 0.995$, 0.1 – 1 µM

$i_{peak\ 1}(\mu A) = -0.071c_{DIP}(\mu M) - 9.78$, $R^2 = 0.954$, 5 – 15 µM

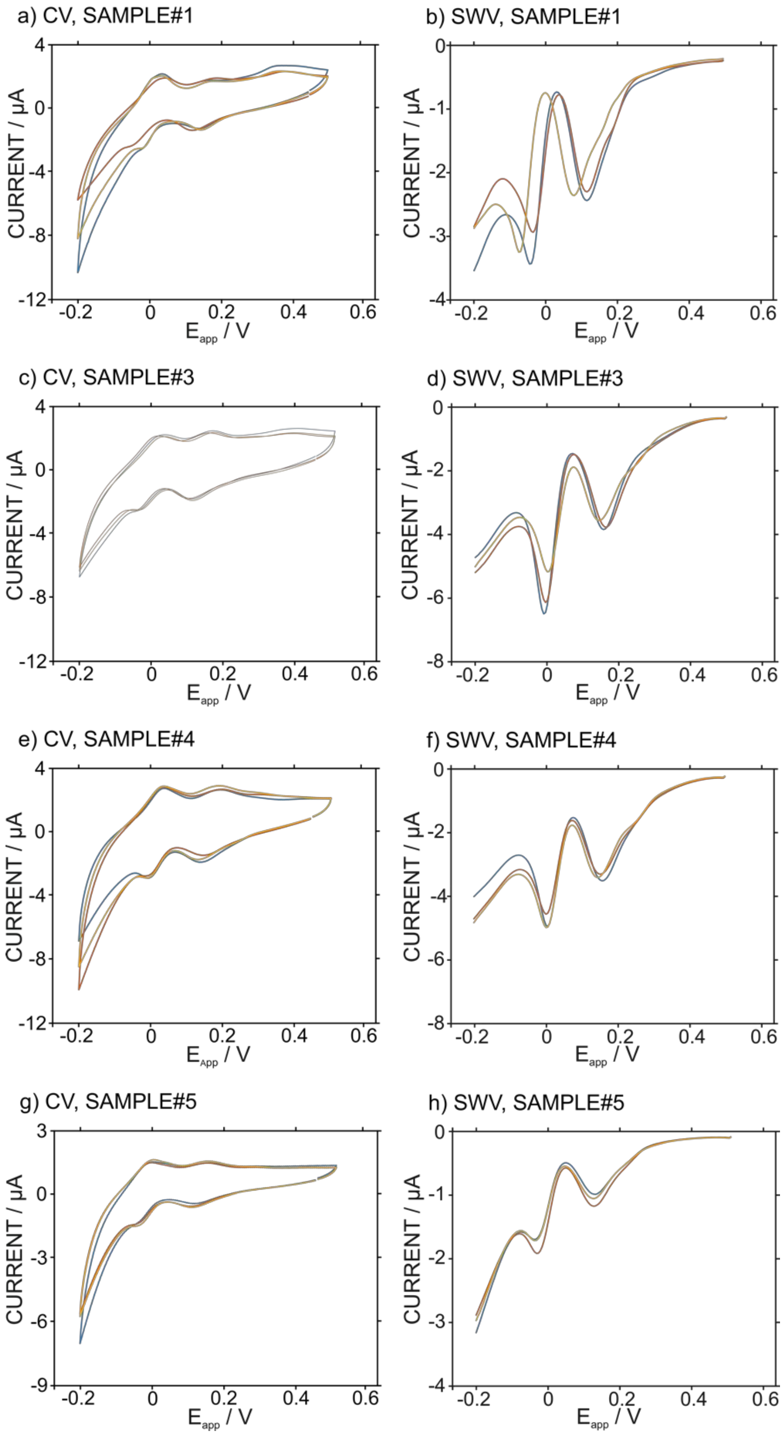

Figure S10. Triplicate voltametric signals (CV and SWV) for seawater samples. (a – b) sample #1, (c – d) sample #3, (e – f) sample #4, (g – h) sample #5. All CVs were measured with scan rate of 50 mV s^{-1}, and SWVs were measured with frequency of 25 Hz, modulation amplitude of 25 mV and potential step of 2 mV.

4. Reference

1. Xuan, X.; Pérez-Ràfols, C.; Chen, C.; Cuartero, M.; Crespo, G. A. Lactate Biosensing for Reliable On-Body Sweat Analysis. *ACS Sensors* **2021**, *6* (7), 2763-2771.
2. Wiorek, A.; Cuartero, M.; De Marco, R.; Crespo, G. A. Polyaniline Films as Electrochemical-Proton Pump for Acidification of Thin Layer Samples. *Anal. Chem.* **2019**, *91* (23), 14951-14959.
3. Wiorek, A.; Hussain, G.; Molina-Osorio, A. F.; Cuartero, M.; Crespo, G. A. Reagentless Acid–Base Titration for Alkalinity Detection in Seawater. *Anal. Chem.* **2021**, *93* (42), 14130-14137.
4. Saji, V. S.; Lee, C.-W. Molybdenum, Molybdenum Oxides, and their Electrochemistry. *ChemSusChem* **2012**, *5* (7), 1146-1161.
